# Measuring the communicative constitution of organization as network formation

**DOI:** 10.1371/journal.pone.0300399

**Published:** 2024-04-09

**Authors:** Kyle Michael Schwing, Jonathan Pitt

**Affiliations:** Virginia Tech, Blacksburg, Virginia, United States of America; The University of Arizona, UNITED STATES

## Abstract

We propose a set of metrics, based upon the four flows theory of the communicative constitution of organizations, to evaluate the emergence of organization in a social network. Using an agent-based model (ABM), we validate that our metrics chart the evolution of partial organizations as the population progresses from complete dissociation to unified allegiance. Our metrics allow the evaluation of organizational strength much more efficiently than previous, context-specific methods. The simulation produces other results consistent with human society, such as stable heterogeneity of structures and organizational figureheads, further validating our results. The ABM of emergent organization incorporates only widely-observed cognitive behaviors and the recognition by agents of group membership, without any cooperation among the agents. The four flows are produced solely by agents biasing their limited communication resources in favor of allies. While reaffirming the centrality of communication patterns to organization, we thus also challenge the minimal conditions required to produce organizing behavior and complex social structures.

## Introduction

Organizations are formed from, and shape, social networks. However, while a rich body of literature discusses the identification of community structures within networks, such methods rarely are grounded in organization theory and the detection of organizational emergence is relatively unexplored. We propose network metrics, derived from the theory of the communicative constitution of organizations (CCO), to test whether organization is emerging and measure its progress. We validate these metrics in the context of an agent-based model that simulates the emergence, through communication, of organization from a state of total isolation. We thus address the call of previous researchers, who criticized that “while the majority of empirical studies in CCO scholarship tend to rely on already well-established and formal exemplars of organizational communication or organization, on the whole they tend to fall short in providing evidence for one of the key assumptions in CCO thinking; namely, that communication predates organization and plays a formative role in organization” [1, p. 489].

To ensure the applicability of metrics to as wide a variety of scenarios as possible, we design a simulation that presumes only nearly-universal cognitive processes and a minimum of awareness or behavioral changes associated with emergent organization. Agents in the simulation do not plan or coordinate their behavior with others; they merely exercise the ability to affiliate themselves with cohorts and prioritize communication from co-affiliates. The emergence of aggregate patterns, despite these restricted circumstances, consistent with CCO predictions for more elaborate and deliberate organizing efforts challenges the minimum behavior required to organize, and if the impact of organizational efforts may be overestimated.

Our study may be framed as an exploration of how social structure emerges from first principles of human cognition, and if this process may be measured in real time. The simulation could represent any group of disaggregated individuals moving toward forming a new apparent social actor, such as a tribe, insurgency, corporation, or other polity. These diverse scenarios all share common elements captured in the simulation; individuals pursue their self-interest and communicate with new associates amid psychological limitations. We do not claim to have captured every process present in real-world examples, but merely to have demonstrated that apparent “organization” may be a spontaneous consequence of basic communication.

These seemingly academic questions about the minimal requirements for “organization” to appear challenge policy assumptions with practical consequences. For example, aspects of law hinge on identifying organizations such as civic affiliations and criminal groups. Similarly, strategic military objectives often are framed at dismantling organizations. Therefore, the ability to objectively define and measure the continued presence of such organizations would seem to be a necessary antecedent to political functioning. Our approach explores potential metrics to meet this need, while also questioning what organizing behaviors are necessary to meet the definition of constituting an organization. If “organizations” exist that lack the capacity for internal cooperation, for shared objectives, or to refine group identity beyond mere existence, but are mathematically indistinguishable from more deliberate and self-aware groups, it challenges the treatment of these collections of individuals as coherent social actors.

This modeling approach also tests the centrality of communication in forming social structures, and if social recognition is the minimum criterion for organization. The simulation tests whether organizations are not just formed by communicating about them, but reside in communication patterns themselves. The simulation also explores how simple mechanisms can produce self-organization and related social phenomena in a complex system, such as stable heterogeneity in social structures and the appearance of leaders.

## Past work

### Communicative constitution of organizations

CCO theory explores how organizations are discursively created as social phenomena. Here, we primarily focus on the “four flows” branch of CCO, which proposes that organization arises as an intertwined process of activity coordination, self-structuring, membership negotiation, and institutional planning [2]. This interpretation of CCO is not only among the most structured, and therefore approachable with quantitative techniques, but also defines communicative acts broadly without presupposing organizational structure [3, 4]. A critique of the four flows definition of organization is that it insufficiently differentiates between formal organizations and spontaneous phenomena, such as markets [5]. In response to these criticisms, the authors of the four flows theory have suggested that if a social movement begins to systematically execute the flows and is socially recognizable as an entity, that it has transitioned to being an organization [6]. As this exchange hints, after the original formulation of CCO a consensus has emerged that there exist partial organizations, which display a limited level of one or more of the flows [7]. The recognition of partial organizations, and later deduction that organization therefore exists along a measurable continuum [3] characterized by variations of the flows [8], are recent developments that have expanded beyond the original framing of the four flows. In this work we focus on the subset of partial organizations that are emergent, with the constraint of being monotonically increasing toward greater levels of organization.

Different approaches to CCO respectively focus on the presence of an organization as an entity, the act of organizing, of the characteristic of being organized [1]. The last framework is most pertinent to this work, as it pushes the boundary of the minimum conditions necessary for organization to emerge. For example, bicycle commuters may display traits of organization, mediated by their collective physical actions, without any central planning or conscious self-identification as a group [9]. Such studies bring focus to discrete act of communication and then their consequences as acts of organization, rather than the reverse [10]. This approach permits phenomena such as partial organizations that are not yet recognized as social actors [11], challenging earlier assertions that organizations are defined by external recognition and deliberate collective action [12].

### Network science

Our study is among the first to explicitly link CCO theory to network metrics. Although previous literature notes the conceptual overlap between CCO and network approaches to modeling human interaction, it mostly focuses on theoretical discussions rather than practical approaches to measuring organization. For example, CCO theorists have proposed a bibliometric-style approach to modeling other communication, defining nodes as communication events and edges as their participants, to illuminate discursive structures [13]. Conversely, network theorists have explored how social networks impact the use of language, and implicitly how communication in turn shapes the network, but have not linked this process to the constitution of formal organizations [14].

Community detection in networks is a major area of inquiry, with a diversity of methods having been proposed to delineate organizational membership [15]. These methods often are validated by comparing case study results to random graphs, and the parameters of a particular community detection algorithm therefore could be construed as a mechanism to measure the degree of organization away from disorder [16]. However, without a grounding in organization theory, it is happenstance whether the structural quirk of a particular networked organization could be interpreted more broadly as a universal measure of organization [17]. Furthermore, most community detection methods treat the existence of an organization as binary, rather than the continuum suggested by quantification of CCO [3]. For example, this rigidness is true even of network methods applied to criminal networks [18], which CCO has recognized as examples of partial organization [19].

Although lacking an explicit measure of this continuum, the emergence of social networks is a topic of frequent inquiry. For example, researchers have examined the communication networks that develop after natural disasters [20], the introduction of new social media forums [21], and even disruptions among non-human animals [22]. Of particular interest to our study is the fact that many of these emergent cohorts lacked central authority or self-awareness as an organization. Intriguingly, models of network formation show how local interactions can produce the appearance of high-level structural organization by producing feedback loops that shape network structure [23]. Even selfish individuals may still produce this emergent organization [24]. The centrality of adaptive learning in response to the behavior of neighbors in these models informs our hypothesis that bandwagon effects may underlie the manifestation of apparent organization. Furthermore, behavior specialization and political structuring have been shown to emerge under similar local feedback loops [25]. These activities resemble CCO flows of activity coordination and self-structuring, an intriguing parallel given that the traditional discussion of the four flows does not anticipate their potential emergence in a complex system from interwoven feedback mechanisms [2].

## Cognitive principles incorporated into the simulation

We develop a novel simulation of social network formation to test whether the four flows of CCO may be artifacts of basic cognitive principles. Exercising parsimony, we limit our simulation to universally-accepted, widely applicable aspects of social cognition that manifest regardless of specific context. These principles are among those frequently cited as potentially key to the bidirectional impact between individuals and the social networks that they form [26].

### Limited communication capacity

Cognitive and physical constraints limit the rate at which people can convey or receive information. This basic experience of all people is grounded in the structure of the brain [27]. Nonetheless, this limitation is sometimes overlooked in models of digital communication. For example, communication limits are a major reason that epidemiology models fail to forecast the diffusion of information in social media: our attention is much more constrained than the myriad points of potential viral infection in the human body [28]. Limited communication capacity directly impacts the number of social ties that a person may maintain, thus shaping the emergence of social structures [29].

### Decaying memory

Many networks formed by cognitive processes gradually erode over time, reflecting the decay of internal memory or its physical artifacts in the environment [30]. Decay in communication networks is particularly prominent and relevant to our study [31]. A variety of mathematical functions have been used to model the decay of individual or collective attention [32, 33], and multiple studies confirm that the rate of decay is dependent on the particular context of a relationship [34, 35]. Regardless of the particular mathematical form used, generally a decaying relationship is modeled as stochastically impacting the likelihood of an event recurring. The bond is then refreshed if recurrence does occur [36]. This feedback loop, preferencing the most frequently-activated network links to be used yet again, is a form of stigmergy. First described as a mechanism for apparent coordination in social insects, stigmergy also has been recognized in the feedback loop between individual action and social norms [37]. Given the ability of stigmergy to produce apparent organization without planning in a wide variety of systems [38], it is a particularly important phenomenon to include in our study of how CCO may emerge from basic cognitive processes.

### Bandwagon effect

The bandwagon effect is the tendency of individuals to adjust their opinion to align with the prevailing attitude of their social environment. It has been described in political science, marketing, and communication studies since at least the 1950s, and explained through a variety of theoretical frameworks [39, 40]. Despite challenges, its existence continues to be upheld in empirical studies [41]. The bandwagon effect has been incorporated successfully into agent-based models of social action, including simulations of spreading unrest or affiliation with a social movement [42, 43]. Here we focus on the bandwagon effect through its role in complex social contagions. Complex contagions on networks are those that require multiple exposures to a stimulus to induce a change [44]. One potential cause of behavioral changes propagating as complex contagions is that the changes are risky actions, and potential adopters wish to see the outcome of their neighbors making the change before taking the leap themselves [45].

### Homophily

Homophily is the tendency of individuals to associate with others that share key traits. It is ubiquitous not only in human networks but also among other species, and evolutionary pathways have been suggested for its emergence amid a wide diversity of contexts [46, 47]. Other studies have shown how a small amount of homophily may restrict opportunities for diversity in social linkages, compounding the apparent magnitude of more deliberate preferences [48]. Ironically, homophily also can cause an innovation to take root within a group of like-minded individuals and incubate before spreading through the network more robustly than the innovation would otherwise [49]. This tendency makes homophily essential to modeling the seeding and diffusion of behavior on a network [50]. Homophily must also be considered because it is often conflated with, and empirically very difficult to separate from, contagion [51, 52]. These two phenomena therefore appropriately are often included together in models of dynamic network formation [53]. Intriguingly, some models demonstrate that these mechanisms and the positive feedback loop between them can yield strongly-differentiated communities in a network [54].

## Model design and hypothesis

Our simulation is an agent-based model, comprising 150 agents that randomly explore a two-dimensional map, encounter one another to form a dynamic communication network, and seek to maximize their individual utility by bidding for assigned roles over several turns, or time steps. Roles are matched on a one-to-one basis to agents at the conclusion of each turn of the simulation, and represent a basic allocation of labor. Roles initially are assigned randomly, and thereafter agents seek to maximize the alignment of their selected role with their internal, static preferences. This aspect of the simulation could be interpreted as the free-market adoption of professions by workers, the assignment of positions to players on a sports team, or the assumption of specialized roles by members of a crowd. For example, it reflects anecdotal descriptions of how organized revolutionary behavior emerged from disorganized mobs of protesters. Participants in such movements later described spontaneously forming groups to undertake specific tasks, such as storming a building, which fostered a sense of belonging and commitment to group endeavors [55]. Our goal is to test whether apparent organization could emerge spontaneously, without any central planning or even individually cognizant attempts to cooperate, if agents are allowed to “feel” this sense of camaraderie.

We test whether homophily-based complex contagion in a network, which causes agents to affiliate with the originator of random messages according to their personal preferences and the disposition of neighboring nodes, is sufficient to change aggregate behavior of the population in ways consistent with the four flows of McPhee and Zaug [2]. Affiliation causes no change in individual behavior, other than increased attention on messages from co-affiliates amid limited communication bandwidth. Initially, each agent has a unique affiliation, and we hypothesize that as the simulation progresses and agents co-affiliate with one another that apparent organization will emerge in the population. Control scenarios prohibit the agents from changing affiliation, and include a scenario in which every agent maintains its unique affiliation and a scenario in which the entire population shares the same affiliation. It is significant to note that agents are equally selfish in both the experimental and control conditions, and always lack any explicit coordination. Our hypothesis therefore posits that the four flows of the communicative constitution of organizations may be artifacts of local communication among agents exhibiting basic cognitive processes.

We set the experimental population to 150, the maximum population size in which all members may share interpersonal relationships without exceeding the capacity of the human brain [56]. We chose this number because it is likely that organization in larger populations would be driven by more complex dynamics, or at least two modalities for population subsets below and above this threshold [57]. We assume that affiliation is a costly behavior, incurring physical or social risk, as defined by Centola [45].

### Simulation design

We review key components of the simulation below. The simulation compromises a series of turns, during which all agents move, form edges with collocated agents, communicate, place bids for roles, and are assigned new roles. The entire population concludes each of these activities before collectively progressing to the next activity. Within each activity, the order in which agents participate is random. The variables and constants of the simulation are summarized in Table 1, and the state variables defining each agent are listed in Table 2. Python code for the simulation is provided in the S1 Appendix.

**Table 1:** Variables and constants of the simulation.

Property	Symbol	Type	Domain	Dimension
Population size	|*I*|	Input constant	N	Scalar
Population	*I*	Random constant	Agents	Set up to size |*I*|
Map length	*L*	Input constant	$N^{+}$	Scalar
Turn number	*n*	Computed variable	$N^{0}$	Scalar
Adjacency matrix	*A* ^*n*^	Computed variable	[0, 1]	Matrix, |*I*| × |*I*|
Neighborhood threshold	*T*	Input constant	[0, 1]	Scalar
Role	$\vec{R}$	Random constant	{1, 2, 3, 4, 5}	Vector, 1 × 5
Broadcast strength	*m*	Random variable	[0, 1)	Scalar
Bandwidth	*c*	Input constant	$N^{+}$	Scalar
Co-affiliate bonus	*b*	Input constant	[0, 1]	Scalar
Edge decay	*d*	Input constant	[0, 1]	Scalar

**Table 2:** State variables of each agent.

Property	Symbol	Type	Domain	Dimension
Location	(*x*, *y*)^*n*^	Computed variable	{1…*L*}, {1…*L*}	Vector, 1 × 2
Neighborhood	*H* ^*n*^	Computed variable	⊂ {*I*}	Set up to size |*I*|
Traits	$\vec{R}$	Random constant	{1, 2, 3, 4, 5}	Vector, 1 × 5
Favorite trait	*v*′	Random constant	$\vec{V}$	Scalar
Assigned Role	${\vec{R}}^{n}$	Computed variable	$\vec{R}$	Vector, 1 × 5
Utility	*u* ^*n*^	Computed variable	[5, 125]	Scalar
Affiliation	*F* ^*n*^	Computed variable	*I*	Scalar

The independent agents and communication links between them may be considered the nodes and edges, respectively, of a network or mathematical graph. This network may be represented as an adjacency matrix, a two-dimensional chart listing every agent along both axes. The value of the matrix at row *i*, column *j* corresponds to the weight of the edge from agent *i* to agent *j*.

The simulation is an abstraction, and therefore variables do not strictly correlate to real-world phenomena. As noted previously, we believe that a population greater than 150 individuals likely would exhibit behavior beyond the scope of the simulation. Nonetheless, if the simulation were used to model an actual event, it would be important to consider the interrelationships of the input constants. For example, the sizes of the map and population should be selected so that the rate at which individuals potentially encounter new collaborators matches the case study. The bandwidth subsequently should be set to the number of meaningful messages that an individual could send or process in that same unit of time, where “meaningful” indicates the potential ability to sway allegiance and would depend on historical context.

### Initialization and network formation

Each agent is located in a square matrix of length *L*, representing discrete two-dimensional space. Any number of agents may occupy the same location. Opposite sides of the matrix are linked, forming a torus and preventing edge effects. At the beginning of the simulation, agents are located randomly in the matrix. At each turn, they move to an adjacent cell in a random cardinal direction.

As agents move, they form network edges with other agents that they encounter in the same location. The adjacency matrix $A_{ij}^{n}=0$ for all agents *i* and *j* at the beginning of the simulation (*n* = 0), and $A_{ij}^{n}=A_{ji}^{n}=1$ if *i* and *j* are collocated after the movement phase of turn *n*. Due to the decay of memory, $0\leq A_{ij}^{n}\leq 1$. Thus, *A* is a weighted, bidirectional, and symmetric network. However, some calculations consider a node’s neighborhood, membership in which is defined in a binary rather than continuous fashion. We therefore define the neighborhood $H_{i}^{n}$ of node *i* at turn *n* as the set of nodes *j* such that $A_{ij}^{n}>T$, where *T* is a constant “neighborhood threshold.”

Every agent has a trait vector constant $\vec{V}$ of five integer values ranging from 1 to 5. Agents are randomly assigned roles, $\vec{R}$, which also consist of five integer values ranging from 1 to 5. The initial values of all $\vec{V}$ and $\vec{R}$ are set randomly at the beginning of the simulation, and remain constant thereafter. The utility of agent *i* at turn *n*, $u_{i}^{n}$, is defined as $\vec{V}\cdot \vec{R}$.

### Communication events

Each turn *n*, after all agents have moved and potentially formed or restored edges with collocated agents, random chatter occurs across the network. In random sequence, agents create and attempt to broadcast messages to all nodes in $A_{ij}^{n}$. As the message propagates, each receiving agent in turn attempts to broadcast the message. We constrain communication such that an agent is unable to receive a message that it has transmitted previously, eliminating echoes in the network. An agent generates a random variable, *m*, each time that it attempts to broadcast, where *m* is uniformly distributed across the range 0 ≤ *m* < 1. Each agent has a limited bandwidth per turn, *c*, which decrements once per message that the agent receives or transmits (regardless of how many neighbors receive the message). Assuming that agents *i* and *j* have remaining capacity, transmission of the message occurs from *i* to *j* if $m<A_{ij}^{n}$. Each time that a message is successfully passed from *i* to *j*, the value of $A_{ij}^{n}$ reverts to 1.

### Affiliation changes

If $m\geq A_{ij}^{n}$, it is possible that the message may still transmit from *i* to *j* if *i* and *j* share an affiliation. The ability of an agent to adopt an affiliation, which biases its receptivity to messages from co-affiliated agents, is the heart of our experiment and our proposition for how apparent organization may emerge. When *i* attempts to transmit a message to co-affiliate *j*, *j* receives the message if $m<(A_{ij}^{n}+b\times \left(1-A_{ij}^{n}\right))$, where *b* is a variable defined by the experimenter such that 0 ≤ *b* ≤ 1.

A received message may prompt an agent to change its affiliation. In experimental conditions, every agent initially is affiliated with itself. An agent imbues its affiliation onto any message that it originates. As the message propagates, a receiving node will consider changing its affiliation to the affiliation of the message in a manner consistent with homophily and the bandwagon effect. Every agent *i* has a “favorite” trait, *v*′,among the five values of $\vec{V_{i}}$, which is assigned randomly at the beginning of the simulation and remains constant throughout the experiment. After receiving a message, agent *i* segments its neighbors $H_{i}^{n}$, defined above, into subsets of agents that share each affiliation *F*, *H*_*F*_. Agent *i* then considers the appeal of joining each cohort through the lens of *v*′:

$$\begin{array}{c} Appeal_{F}=\sum_{h\in H_{F}}4-\left|v_{h}^{\prime }-v_{i}^{\prime }\right| \end{array}$$

Agent *i* compares the appeal of its current affiliation to that of the message’s affiliation, and chooses the affiliation *A* that has the greater appeal. Note that an affiliation propagates through the network based on the individual preference and local neighborhood of each node, not the characteristics of the agent that originated the message. Therefore, for example, it is possible for agent *i* to originate a message to agent *j*, prompting *j* to affiliate with *i* before passing the message on to *k*, which also affiliates with *i* due to $\vec{V_{j}}$, even though *k* would not have been prompted to change its affiliation based on $\vec{V_{i}}$. Also note that whether an agent changes its affiliation due to a message is independent of whether the agent transmits the message further along the network.

In the control scenarios, agents may not change their affiliation. There are two controls; in one the agents maintain unique affiliations, and in another they all have the same affiliation. We hypothesize that these two scenarios bound behavior between populations that respectively cannot organize and are fully organized, and that emergent organization will appear in the region between the two extremes.

### Bidding and edge decay

After communication events have exhausted the bandwidth of all agents, and potentially changed the value of *A*^*n*^, the agents bid for roles $\vec{R}$. Agent *i* only considers roles currently assigned to members of its neighborhood *H*_*i*_. It ranks its preferred roles to maximize the utility of each potential role, as defined above, in the context of $\vec{V_{i}}$. The agent assigns equal bids of minimum value to all roles assigned outside its neighborhood. After all agents have ranked their preferences, the simulation assigns each role to the agent that ranked it highest. If multiple agents gave the role equal ranking, it is assigned randomly among those agents. After an agent has been assigned a role, its rankings are voided so that it no longer competes for other roles.

After all agents have been assigned new roles, all values of *A*^*n*^ decrease by a fixed variable *d*, defined such that 0 ≤ *d* ≤ 1. This is the final step of each turn *n* before the simulation progresses to the next turn. The simulation will execute an arbitrary number of turns, set by the experimenter, before concluding.

## A network analysis approach to CCO

We define the four flows of CCO [2] in terms of network phenomena to measure partial, emergent organization in the simulation data. This novel quantification of CCO is only the fourth study to propose a measurement of CCO flows [3, 58, 59]. Of particular significance, a network-based approach avoids the laborious, context-dependent definition and coding of data that prior efforts to quantify CCO depended upon [58, 59]. Our method provides a quick and efficient means to track the emergence of an organization, potentially in real-time, limited only by the availability of basic observations such as network communication data. This efficiency also enables us to take more measurements than previous methods, yielding finer resolution when examining shifts over time.

The most difficult challenge of defining CCO flows in network terms is separating the flows from an a priori definition of the organization. For example, institutional positioning is how an organization creates a social identity, differentiating itself from other organizations. How, therefore, can institutional positioning be defined without assuming the existence of the organization that is the subject of inquiry, nor any other organizations potentially present? Community detection in networks is a robust field of study but, due to the emergent nature of the organizations under scrutiny, community membership would need to be calculated after every turn. The vast majority of algorithms used to detect and define communities within networks are too computationally intensive to execute so frequently [60]. In our simulation, it would be possible to examine some organizational membership data directly, particularly the affiliation of the agents. However, this data is only readily available in the abstract context of the simulation, and would be difficult to collect in real-world scenarios without surveys or other intensive methods. The gap between theoretical constructs and feasible observation is a recurring issue when studying the propagation of attitudes on networks [61].

We therefore propose measuring the anticipated outcome of the CCO flows, rather than the execution of the flows themselves. For example, we explore what consequences would result from institutional positioning that reifies the distinction among organizations. Our methods focus on the transaction network of communication events, and disregard the hypothetical content of messages. Several of our definitions are informed by the observation that an organization is a social mechanism for reducing uncertainty and increasing efficiency [3, 59], and that we therefore expect the rate of change to vary as an organization matures.

The metrics described below have emerged from an iterative cycle of candidate testing and modification, and are summarized in Table 3. Our bias is toward simple rather than complex metrics, for the sake of universality and speed of computation. This is not an exhaustive list of potential definitions of the CCO flows in terms of network data, but as demonstrated below, is sufficient to measure the emergence of organization from a totally unaffiliated state.

**Table 3:** Definition of CCO flows as phenomena among networked agents bidding for roles.

CCO Flow	Network Metric	Explanation
Activity coordination	|*r*^*n*^ − *r*^*n*−1^| where *r*: *I* → *R*	Number of agents that change assigned roles between turns
Self-structuring	$P(\bar{ik}|\bar{ij}\cap \bar{jk})$ $\bar{ij}$ when $A_{ij}^{n}>T$	Percentage of potential network triangles closed
Membership negotiation	∑_*i*∈*I*_|*H*^*n*^△*H*^*n*−1^|	Total number of changes to neighborhood membership lists
Institutional positioning	$\frac{1}{\left|I\right|}\sum_{i\in I}\frac{1}{\left|J\right|}\sum_{j\in J}{\left(A_{ij}-\bar{A_{i}}\right)}^{2}$	Variance of network edge weights

### Activity coordination

Activity coordination is the tactical assignment of tasks to members of an organization. In this work we interchangeably use “role” and “task” to indicate a potentially one-time assignment undertaken by an agent, not a routinized behavior. The algorithm assigns tasks to agents each turn to ensure a one-to-one pairing, a limitation that would be achieved in the real world by physical constraints. As discussed previously, the model assumes no central coordinating authority is present in the population.

We assume in the context of an unchanging environment that a more mature organization is more Pareto efficient than a less-developed one [62], and that since the more-mature organization is closer to an optimal state its members will make fewer adjustments to maximize their utility. We therefore hypothesize that changes in assignment will grow less frequent as the level of organization increases. We measure activity coordination directly from our simulation data as the number of agents that are assigned new roles each turn. Because we do not assume the presence of coordination, this metric is a measure of aggregate, individual behavior. As discussed above, the metric is a measure of the predicted, observable consequence of a CCO flow rather than the flow itself. Although role assignments are not normally part of communication network data, we believe that they still fit our requirement that selected data be readily observable in the real world. For example, such data is inferred whenever task specialization is said to emerge in a society.

### Self-structuring

Self-structuring is the establishment of strategic patterns of behavior through relationships within an organization. We conceptualize self-structuring as the enrichment of network links, the habitualization of channels of communication as protocol emerges. The closure of social triangles, i.e. the increased likelihood that *i* will communicate with *k* if *i* and *k* already both communicate with *j*, are a classic indicator of a richly-developed social network and will drive community formation [63]. We therefore propose using the percentage of closed triangles as a measure of self-structuring:

$$\begin{array}{c} P(\bar{ik}|\bar{ij}\cap \bar{jk}) \end{array}$$

We define $\bar{ij}$ to exist at turn *n* when *i* and *j* are neighbors, or $A_{ij}^{n}>T$. We note that this formulation is identical to the global clustering coefficient [64].

### Membership negotiation

Membership negotiation establishes the rights and responsibilities of membership in an organization, separating those who belong from those who do not. Without querying the nodes for explicit statements of membership, which may be overlapping and in flux, we can only measure the consequences of increasingly distinct membership. For example, as membership becomes starker, agents may be expected to interact more frequently with members of their same cohort than those outside it. This fluid notion of membership as being defined by changing contributions to a group’s endeavors is consistent with recent developments in CCO [65]. We use the neighborhood threshold to define the subset of agents with which an agent interacts most frequently, and examine changes in the membership of this set.

### Institutional positioning

Institutional positioning differentiates organizations from the surrounding social environment, particularly other organizations. As noted above, this construct presents a challenge for measurements that cannot assume the existence or boundaries between organizations. However, as institutional positioning strengthens, it increasingly differentiates how an agent interacts with one social cohort versus another. In network communication data, this trend could be measured as increasing variance in the distribution of edge strengths between a node and the rest of the population. Although explicit edge strengths are a synthetic artifact of our simulation, they are a proxy for real-life data such as the frequency of communication between two particular agents over time.

## Results

The simulation data distinguishes clearly among the experimental and two control scenarios, validating our proposed CCO-inspired metrics as a method to distinguish emergent organization, the absence of organization, and robust organization. In the experimental case of emergent organization, the data for the three metrics that measure the degree of order in the system all progress from the control state of the unaligned, unorganized population to the opposite control state of the fully-aligned, robustly organized population. The fourth metric, the variance of communication network edge strengths as a measure of institutional positioning, reveals the unique establishment of multiple network cohorts within the experimental scenario.

We conduct the baseline simulations with a population of 150 agents interacting in a 10x10 grid. The maximum communication bandwidth of each agent is set to six messages, edge weight threshold for inclusion in a node’s neighborhood is 0.5, and all edges decay at a rate of 0.8 per turn. The “bonus” *b* granted to messages from co-affiliates is set to 1. 50 turns are sufficient to reach steady-state trends, and the results provided below are the average of at least 50 trials per experiment.

Cross-comparing multiple 50-trial runs verified that our metrics provide consistent results with this sample size. Interestingly, our four metrics are more stable than other potential alternatives. For example, the total utility of the population appears to be very sensitive to random initial conditions, such as agents’ first assignments to roles and geographic positioning. The averages of two separate 50-trial runs appear significantly different despite identical input conditions, suggesting that this aspect of the simulation may be a chaotic system [66]. Increasing the number of trials to 250 fails to yield more coherent results, buttressing this assessment.

### Role and affiliation changes

**Fig 1:**
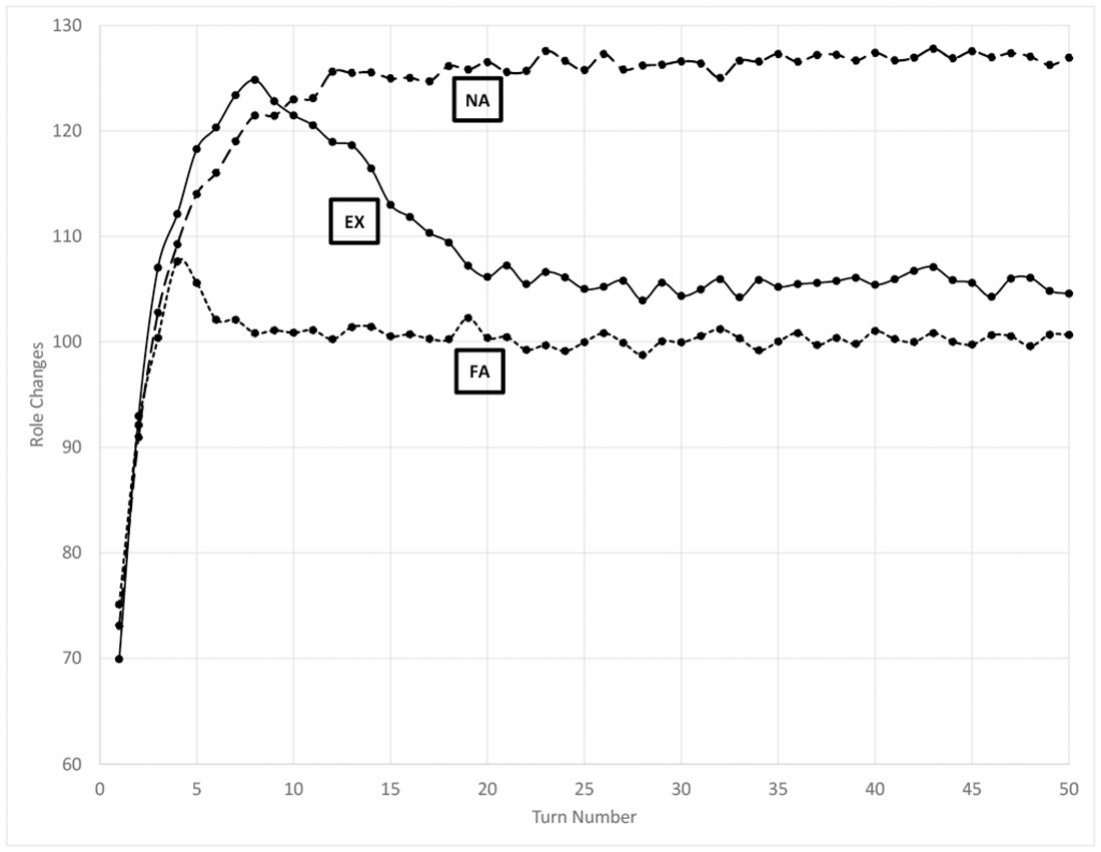
Number of times an agent changes roles in each turn of the simulation under baseline conditions. EX = experimental scenario, NA = non-aligned control scenario, FA = fully-aligned control scenario.

The number of role changes that occur in each turn of the simulation is shown in Fig 1 for the experimental and two control scenarios. As seen in the figure, after an initialization period of approximately 12 turns both control scenarios reach a steady state, but at a greater value for the non-aligned than the fully-aligned control. During this initialization period, agents are moving and establishing connections with one another that increase the size of their neighborhoods, and hence the pool of tasks for which they bid. Steady state is reached when the rate at which new bonds are formed equals the rate at which previous bonds decay below the neighborhood threshold.

Co-aligned agents are more likely to communicate than non-aligned agents, and each communication event renews the strength of edges between agents. Therefore, the predicted size of an agent’s neighborhood increases with the number of co-aligned agents in the population. Neighborhood composition fluctuates throughout the simulation, and can be considered a moving window of what other agents and roles an agent considers for certain decisions. Since this window is larger for the fully-aligned control scenario, it is logical that agents’ bids for preferred roles would be more stable, and the number of role reversals would be less, in the fully-aligned than the non-aligned control scenarios. In other terms, greater information asymmetry in the non-aligned control scenario creates a more dynamic market for roles than exists in the more consistently-informed, fully-aligned scenario.

**Fig 2:**
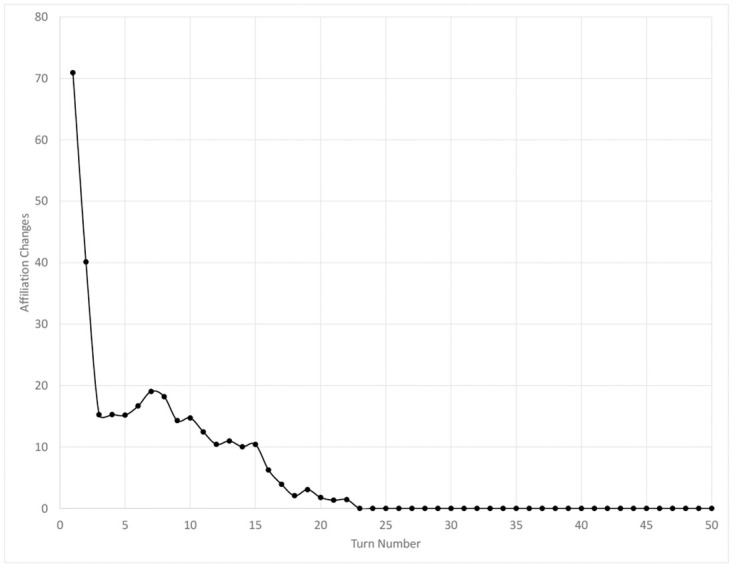
The number of affiliation changes per turn in the baseline experimental scenario.

As the experimental scenario progresses, the random convergence of message threads from the same originator causes affiliations to align locally. The bandwagon effect drives a positive feedback loop that then causes these islands of co-affiliation to grow quickly. Fig 2 demonstrates that the rate at which affiliation changes occur decreases quickly after the simulation begins, with the exception of a brief uptick at approximately turn 7, and reaches zero at turn 23. Experiments show that two stable cohorts emerge, each aligned to a particular affiliation, between approximately one- and two-thirds the population in size. These cohorts share no enduring edges above the neighborhood threshold; any links that emerge due to co-location erode away due to the preference given to co-affiliate messaging amid limited communication capacity.

The transition of the experimental scenario in Fig 1 from paralleling the behavior of the non-aligned to the fully-aligned scenarios is due to this growth in co-aligned cohorts in the experimental scenario. The experimental scenario parallels, rather than asymptotically approaches, the fully-aligned data because the experimental scenario population is divided into two large cohorts at steady state. Note that this paralleling begins at approximately turn 23, which also is when affiliation changes cease. Although members do not move between the two cohorts, it is still possible for messages to be exchanged between them and members of each cohort to be in the other’s neighborhood. Agents therefore can “discover” roles that affect their bidding preferences, increasing the churn of assigned roles and keeping the baseline rate of role changes slightly higher for the experimental versus fully-aligned control condition.

### Percent triangles

**Fig 3:**
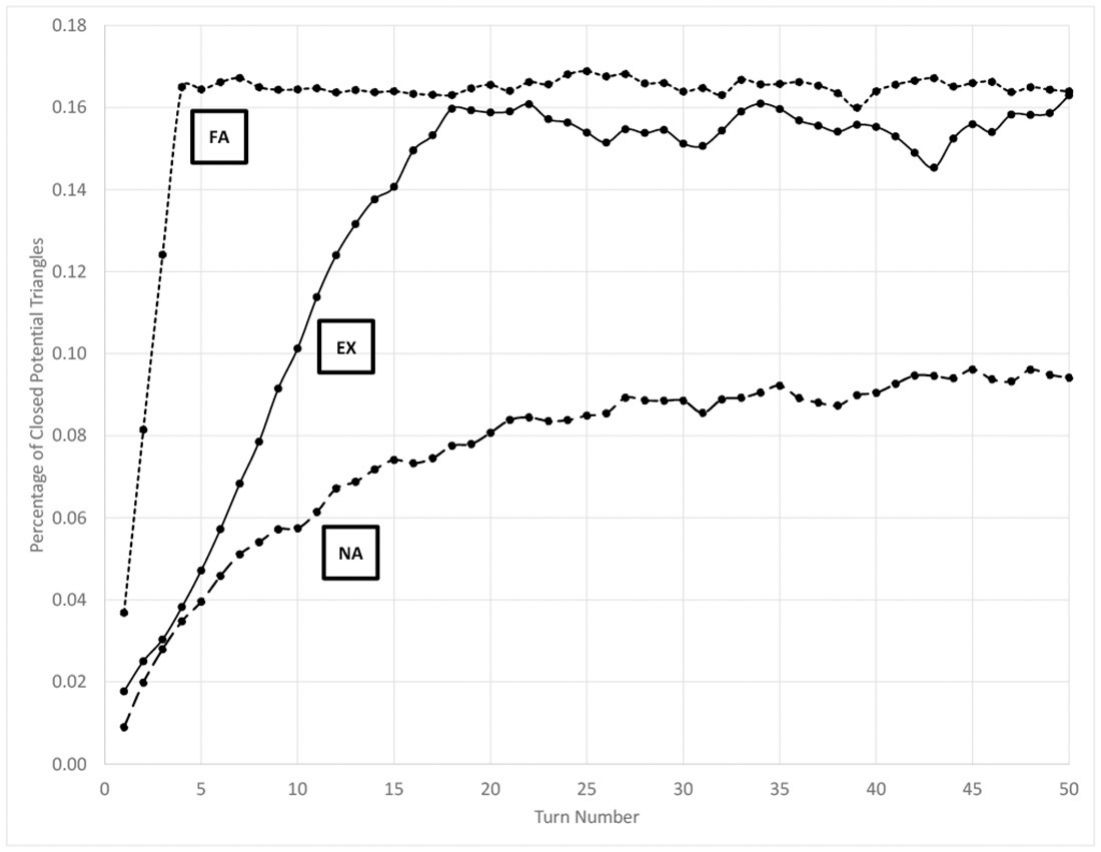
Percentage of closed potential triangles at each turn in the network of communication links that exceed the neighborhood threshold. EX = experimental scenario, NA = non-aligned control scenario, FA = fully-aligned control scenario.

The percentage of potential triangles, links above the neighborhood threshold forming between agents *i* and *k* if *i* and *k* already both link to *j* as defined above, is shown in Fig 3 over the duration of the simulation. Similar to the role change data, the experimental scenario first approximates the non-aligned control scenario before evolving to parallel, but not reach, the fully-aligned control scenario. The inflection in the experimental data again occurs at approximately the point at which agents have stopped changing affiliation.

All agents are inclined toward forming triangles because of the spatial aspect of how some edges are formed; if *i* and *k* both recently encountered *j* nearby then odds are greater that *i* and *k* also will encounter one another than would be true otherwise. However, agents also are more likely to form triangles as the relative concentration of co-affiliates rises because of the increased likelihood of successful communication events between co-affiliates. This boost increases the overall number of edges above the neighborhood threshold, including in triangles. Therefore, the fully-aligned control scenario more quickly reaches a higher equilibrium percentage of triangles than the non-aligned control scenario, which grows more slowly to a lower steady-state value.

### Neighbor changes

**Fig 4:**
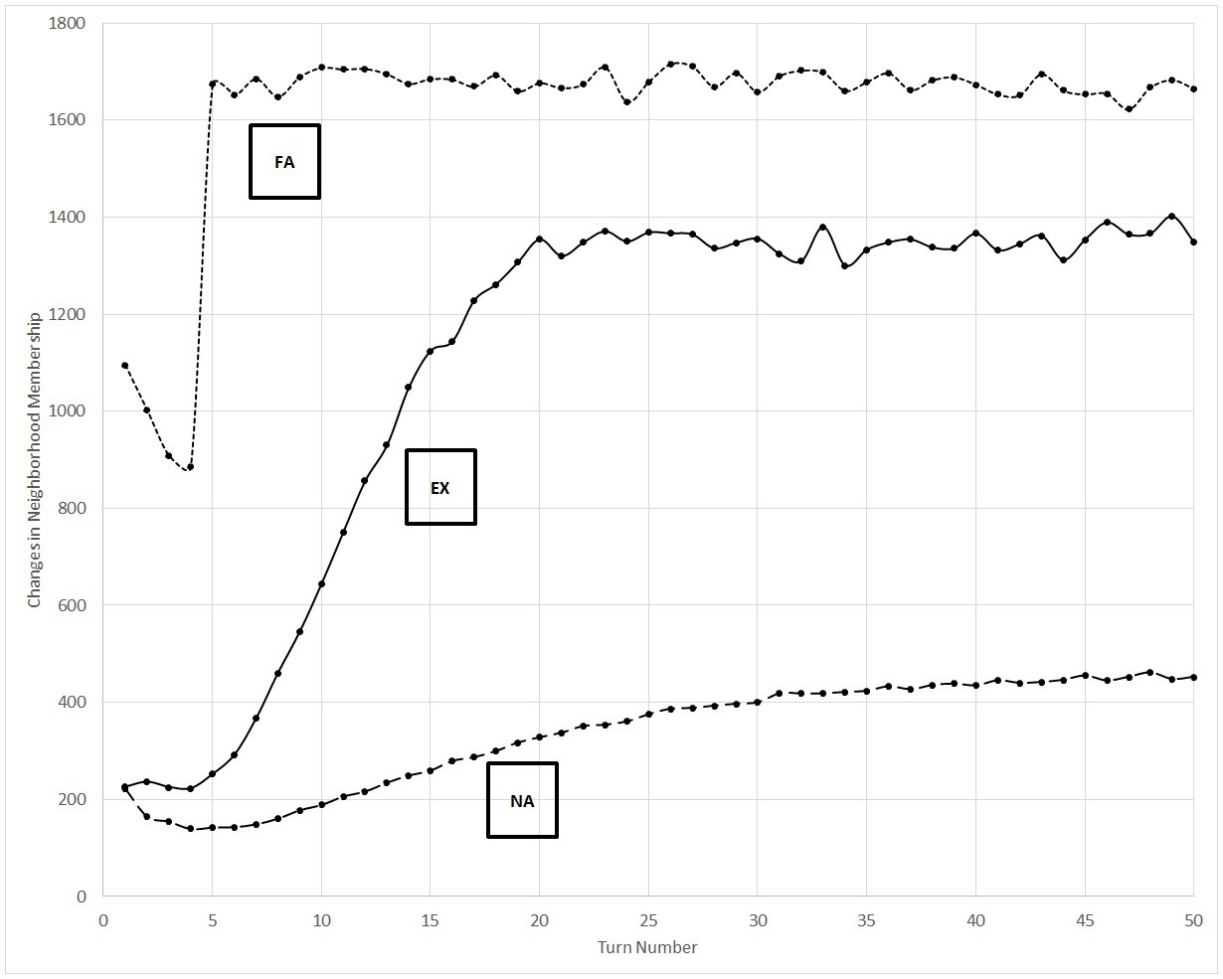
Collective change in membership of the neighborhoods of the nodes since the previous turn. EX = experimental scenario, NA = non-aligned control scenario, FA = fully-aligned control scenario.

Fig 4 displays the total number of changes to individual neighborhoods, caused by agents building or losing edges greater than the neighborhood threshold, since the previous turn. This behavior is modulated by the increased likelihood of successful communication among co-affiliates, and therefore the experimental scenario again passes from resembling the non-aligned control scenario to the fully-aligned control scenario.

An interesting trend across all three scenarios in Fig 4, and most pronounced in the fully-aligned control scenario, is the initially decreasing rate in the number of changes of neighborhood membership before a sharp increase between turns four and five. We note that this inflection approximately coincides with the inflection in the rate of affiliation changes shown in Fig 2. The turn number at which the inflection occurs likely is an artifact of the decay rate of network edges and the neighborhood threshold. In this instance, the decay rate is 0.8 and it takes four additional turns after an initial link is formed for the link to erode below the neighborhood threshold, if a successful communication event does not occur between the two nodes, because 0.8^4^ = 0.41 < 0.5. Thus, the apparent sharp increase in the number of changes to local neighborhoods at turn five is due to the exit of nodes with which links were formed at turn one, a loss rate that remains constant for the control scenarios as the simulation proceeds.

### Edge weight variance

**Fig 5:**
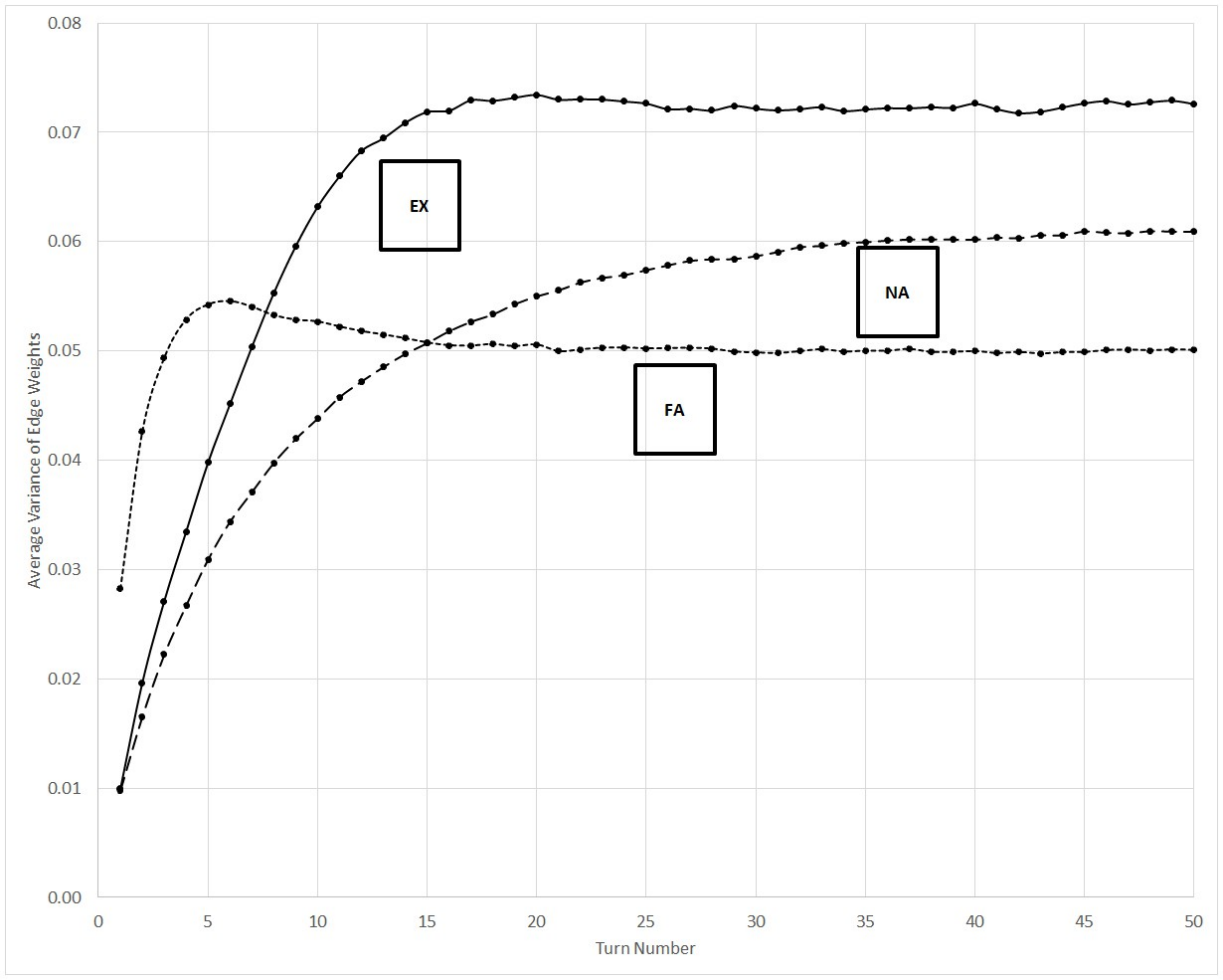
Average variance of set of edges for each agent. EX = experimental scenario, NA = non-aligned control scenario, FA = fully-aligned control scenario.

The average variance of the edges of each agent are shown in Fig 5. This value may be defined as

$$\begin{array}{c} \frac{1}{\left|I\right|}\sum_{i\in I}\frac{1}{\left|J\right|}\sum_{j\in J}{\left(A_{ij}-\bar{A_{i}}\right)}^{2} \end{array}$$

where $\bar{A_{i}}$ is the average value of all edges in the adjacency graph *A* for agent *i*.

This metric tracks differentiation among agents, and therefore follows a different pattern than the previous three metrics. The fully-aligned control scenario contains the least diversity, and therefore has the lowest average variance. After an initial period in which edges grow according to random differences in spatial location, the ubiquity of communication among co-affiliates dominates behavior and the variance of edge weight values quickly reaches a steady state. In contrast, edge weight in the non-aligned control scenario is driven by collocation, and the random walk of the agents continues to generate a greater diversity among the weights of these bonds.

The variance of edge strengths is greatest for the experimental condition. As discussed previously, stark differentiation develops within the network structure as the simulation proceeds. This heterogeneity yields contrasting sets of relationships for an agent, in which it is linked much more strongly to one cohort than another. The edge variance metric tracks and summarizes this distinction among cohorts. Note that the metric reaches a steady state at approximately turn 17, as affiliation changes become very rare in Fig 2.

### Perturbation analysis

Our results appear robust to changes in the input variables, as described below. This consistency is significant, because it buttresses the validity of using these metrics to detect the four flows of CCO in a variety of circumstances.

#### Population density

We vary the size of the matrix in which the agents interact. The population of agents is consistently 150, so altering the matrix size effectively changes the population density of the simulation and the frequency with which agents encounter one another during their random walks. For example, decreasing the population density by increasing the size of the matrix slows the rate at which variables driven by chance encounters occur. All four metrics in the experimental and non-aligned control scenarios take longer to reach equilibrium, and the values of equilibria decrease as population density decreases. In contrast, the fully-aligned control scenario reaches its equilibrium values in the same number of turns regardless of the size of the matrix, albeit at lower steady-state values as population density decreases. This difference may occur because equilibrium in the fully-aligned control scenario is dominated by the rate at which co-affiliate communication bonds decay below the neighborhood threshold, rather than spatial interaction. Interestingly, if population density increases, eventually the increasing likelihood of spatial interaction overpowers the preferential communication toward co-affiliates. Rather than bifurcating into two groups, agents in the experimental scenario align on a single affiliation and the experimental data trends toward the fully-aligned control scenario data.

#### Communication capacity

The maximum number of communication events that an agent can participate in per turn cannot be set less than one for the simulation to function. The steady-state values of the metrics decrease with this variable. This shift occurs because the rate of communication counterbalances the edge decay rate to drive the position of these equilibria. A lower communication capacity tends to diminish the observable gap between the three scenarios and, if sufficiently low, can cause the difference between the steady-state values of the experimental and fully-aligned control scenarios to fall within the range of statistical variation. These observations support the conclusion that the phenomena under consideration are driven by communication, and that decreasing communication tends to squelch their impact. Notably, however, the impact of this perturbation appears to have a limit. After a certain threshold, raising the number of permitted communication events has marginal impact on the number of role changes per turn, particularly for the fully-aligned control scenario. We suspect this result may be because the agents’ awareness of available roles has become saturated, after which reassignment of roles is due to the stochastic nature of the bidding process.

#### Edge decay rate

As discussed previously, a smaller value of the decay variable *d* shifts inflection points earlier due to the more rapid rate of decay below the neighborhood threshold after a successful communication event. Decreasing the value of *d* also increases churn on the network. For example, a lower value of *d* increases the steady-state value of the rate of neighbor changes, while decreasing measures of stability such as the percentage of triangles formed and variance of edge weight.

At very large values of *d* that approach 1, making edge decay negligible, the plateau in the rate of role changes in the fully-aligned control scenario is shifted so late that it occurs after the non-aligned control scenario data has reached steady state. As in the baseline scenario of Fig 1, the fully-aligned control and experimental scenarios approximately parallel the non-aligned control scenario until the first two scenarios begin to plateau. Therefore, the steady-state value of this metric for these scenarios is very similar to the values for the non-aligned control scenario at all times. This output suggests that in the context of the simulation, the decay of communication links is required to give the appearance of increasing activity coordination with greater organization.

As *d* decreases, the distinction between the experimental and fully-aligned control output diminishes for most metrics. A smaller value of *d* and faster edge erosion emphasizes more recent events which, amid increasing co-affiliation in the experimental scenario, more closely resemble those in the fully-aligned scenario than past events. The exception to this trend is that edge weight variance grows more distinct between the experimental and fully-aligned scenarios as *d* decreases. The more rapid decay of bonds formed by co-affiliate communication gives the fully-aligned control a greater variance of edge strengths, more similar to the non-aligned control. The variance within the experimental scenario, meanwhile, remains high due to the bifurcation of the population into two cohorts of co-affiliates.

#### Neighborhood threshold

The decay variable *d* and the edge weight threshold *T* for agents to include one another in their neighborhoods share a special relationship, because if *d* exceeds *T* neighborhoods cannot form at all. Furthermore, changes in *T* often have an effect opposite to a corresponding change in *d*. For example, if *T* decreases, neighborhoods expand and the number of turns that a new edge is included among them increases, shifting inflection points to the right. This effect contrasts decreasing *d*, which shifts inflection to the left. Decreasing *T* causes the steady-state number of neighbor changes per turn to decrease while the likelihood of triangles forming among neighbors and the rate of role changes increases. However, the impact of varying the neighborhood threshold on outputs is dampened in comparison to the impact of varying *d* by an equal but opposite amount. Interestingly, varying *T* has no effect on the average variance of edge weights.

#### Co-affiliate communication

The core difference between the dynamics of the non-aligned control scenario and the experimental and fully-aligned control scenarios is the boost in communication from co-affiliates, *b*. Therefore, changes induced by varying the value of *b* merit special attention, and differentiate regimes within the data.

When *b* is set to zero, the boost is nullified and the outcomes of all three scenarios are identical. However, even a small increase in *b* from 0 to 0.2 quickly distinguishes the fully-aligned from the non-aligned control scenario according to most of the general patterns in key metrics discussed above. These trends persevere as *b* further increases to 1.0, but only grow more distinct as differences in steady-state values increase for some metrics. For example, the steady-state value of the rate of changes in neighborhood membership increases with *b*, but the steady-state percentage of potential triangles within those neighborhoods essentially remains constant. This contrast potentially is because any non-zero value of *b* permits non-local communication, but equally impacts the likelihood of any legs of the triangle forming and thus does not affect the conditional probability of the third leg emerging given the presence of the first two.

**Fig 6:**
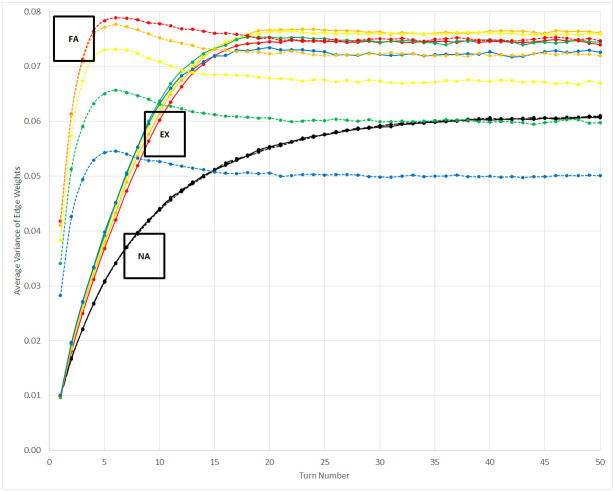
Average variance of set of edges for each agent as a function of *b*. The value of *b* increases from 0 to 1.0 in increments of 0.2, and data are shown in red for b = 0.2, orange for b = 0.4, yellow for b = 0.6, green for b = 0.8, and blue for b = 1.0. Changes in *b* have no effect on the non-aligned control scenario, which coincides with b = 0 for the other two scenarios. EX = experimental scenario, NA = non-aligned control scenario, FA = fully-aligned control scenario.

The measure of edge weight variance, and thus heterogeneity in relationships, is greatest in the fully-aligned control scenario when *b* is set to a small, but non-zero, value. As *b* approaches 1, the steady-state value of the variance decreases because communication becomes more consistent —when *b* equals 1, all attempts at communication succeed and the pertinent network edges are reset to 1. However, in the experimental scenario the steady-state value of the variance peaks when *b* equals 0.5. In general, a greater value of *b* tends to exaggerate the level of diversity or lack thereof among the affiliations of the population. As a result of these mixed effects, differentiating the scenarios at steady state can be difficult for some values of *b*. The relationship of this metric to *b* for each scenario is shown in Fig 6. Before steady state in the experimental scenario, when many affiliations are present in the population, a greater value of *b* produces a slightly greater variance by increasing the boosted effect of co-affiliate communication versus communication with non-affiliates. However, as discussed previously, only two affiliations remain at steady state. At that point, the impact of increasing *b* on creating less variation amid bonds of co-affiliates is counterbalanced by its increasing tendency to distinguish between cohorts of shared affiliation. When two such cohorts are present, the maximum overall variance in network edges occurs when *b* is 0.5.

## Discussion

The simulation outcomes support our core hypothesis, that a group of actors may appear to constitute an organization, exhibiting collective behavior consistent with the four flows of McPhee and Zaug [2], without any central planning or coordination. Merely providing agents a mechanism to affiliate with a group, recognize in-group and out-group members, and even mildly bias communication toward co-affiliates is sufficient to produce phenomena in the aggregate communication network that suggest social organization. For example, these patterns emerge even for very small values of the communication boost *b* provided to co-affiliates.

In essence, we have demonstrated how local or “micro”-scale activities can generate the ephemeral “macro”-level perspective that an organization exists [67]. Our approach, a model of interacting agents that generate a dynamic network, is a natural continuation of prior theories that organization emerges from networked computation [67]. Our outcome is even more remarkable given the simplicity of the agents, which forego many behaviors often assumed to underlie organization [68]. Agents do not act altruistically toward co-affiliates, trust information more from co-affiliates than outsiders, or consider the guidance of an active leader.

Our results do not refute the existence of the four CCO flows, but rather demonstrate that they may be evidence of organization existing rather than a deliberate act of organizing. The flows become the signs and symptoms of organizing, rather than the original mechanisms through which organization is achieved. We have reinforced the centrality of communication to the emergence of organizations, and demonstrated a mechanism through which organization can in turn affect communication by biasing the allocation of limited attention. Organization and communication thus form a feedback loop, and the simulation results suggest that any natural heterogeneity in communication patterns could almost spontaneously cause opposing organizations to form.

### Emergent organization

Our simulation data demonstrate that network data can differentiate a fully-organized population from a non-organized population, respectively represented by the fully-aligned and non-aligned control scenarios. Furthermore, as organization emerges in the experimental scenario the collective behavior of a population evolves from that of the non-aligned to the fully-aligned states. The biasing of communication toward co-affiliates acts as a centripetal force, coalescing disaggregate agents into apparent organizations. The gap between the steady-state values of several metrics for the experimental and fully-aligned scenarios reveal the bifurcation of the population of the experimental scenario into two mutually distinct cohorts with minimal communication between them.

Using differences in the absolute values of the simulation data to detect emergent organization in the real world, however, may be difficult without the availability of data from alternative scenarios for comparison. We therefore highlight trends that distinguish emergent from established organization, such as that seen in the fully-aligned control scenario and experimental scenario after affiliation bifurcation has occurred. For example, note that data from these two latter states in Figs 1, 3, 4 and 5 is mostly constant, while non-horizontal trend lines reveal the emergence of organization earlier in the experimental scenario.

### Measuring the four flows of CCO

The boost given to messages from co-affiliates fosters links between agents that may not encounter one another as they wander the spatial map of the simulation. These edges increase their number of communication partners, giving each agent access to more information at one time. In the context of limited communication bandwidth, the boost also functionally preferences edges with co-affiliates versus the general population, reinforcing division among cohorts of differing affiliation.

The combined effect of a larger, more stable group of partners causes bids for roles to be more stable. Thus, as shown in Fig 1, more efficient activity coordination appears with greater levels of organization, even though no coordination has occurred other than a willingness to listen and gain situational awareness. Similarly, decreasing the comparative effect of recent co-location on communication increases the likelihood of triangles closing in the network. This richer self-structuring is not the result of strategic organizational alignment or even habituated communications, but rather the overall greater level of communication among co-affiliates versus non-affiliates.

Increasing the concentration of co-affiliates in the population increases the rate of membership changes in an agent’s neighborhood, as shown in Fig 4, because fresh members are continuously added from the pool of co-affiliates. This result is somewhat surprising because, if organization is a mechanism for entropy reduction [3], greater levels of organization may be expected to yield more stable membership through the CCO flow of membership negotiation. However, this strict definition of membership negotiation is a behavior of an organization rather than an individual, and as Fig 2 demonstrates, the membership of each co-affiliate group stabilizes as organization emerges in the experimental scenario. (Recall that we deliberately did not include affiliation in our metrics, since its observation in real-world data would require the a priori identification and definition of potential organizations.) The neighborhood change data demonstrates that changes in personal relationships successfully distinguish between states of non-organization, robust organization, and emergent organization, and that this metric inspired by a CCO flow is a valid means to detect organization. Incidentally, the notion that affiliation with an organization could broaden an individual’s social circle is consistent with common experience. Military enlistment is frequently associated with social integration [69], and other organizations have brought together otherwise disparate groups [70].

The average variance of edge weights is unique among the metrics because the steady-state value of the experimental scenario is greater than that of either control scenario, and the non-aligned is greater than the fully-aligned control scenario. These results are expected, since this variance is a proxy measure of the CCO flow of institutional positioning, and it is difficult to strongly differentiate an organization without the presence of other robust organizations. The variance of all edges therefore becomes a measure simultaneously of both cohesion and diversity in the network; it is greatest when the difference between two groups is most sharply evident.

### Validity of results

The validity of our conclusion, that the network-based definitions of the four CCO flows measure organizational emergence, depends on the ability of the simulation design to permit alternative outcomes. One such outcome would have been if the four metrics did not show consistent trends across randomized experiments. As discussed previously, we discovered that alternative metrics, such as the total utility of the population, behaved chaotically rather than converging. Another alternative outcome would have been if the experimental scenario data had abruptly transitioned between the non-aligned and fully-aligned control scenarios, without exhibiting the gradual shift that suggests an emergent organization may be detected in real-world scenarios before it has fully matured. Note that some phenomena, such as the effect of the co-affiliate communication boost *b*, were associated with such sharp transitions. It was also hypothetically possible that any of the selected metrics did not distinguish between the control scenarios. As noted above, this was observed under certain conditions, such as a very low communication capacity.

The conclusions of the study also rely upon the validity of its core assumptions. These premises are that humans experience cognitive and communication limitations, can observe markers of group affiliation, and will bias their limited communication capacity toward members of their in-group. We believe that it is readily apparent that humans have limited memory and communication capacity. However, it is possible to imagine scenarios in which our second assumption, that markers of group affiliation are observable, is challenged. For example, an anonymous online forum may prohibit users not only from revealing explicit markers of affiliation, but also from observing the interactions of users with other members and the implicit affiliations that they may suggest. Our simulation predicts that under these conditions, the communication network structure of the users would not develop indicators of organization. Future research could invalidate this prediction. However, note that such a discovery would not invalidate our overall conclusion that basic cognitive processes can produce the four flows of CCO, but rather would just further reduce the minimum set of such processes necessary to produce the appearance of organization.

Our third assumption, that communication is biased toward members of the in-group, is central to our results. Its significance infers that this communication bias may be the root cause of indicators of organization. The bias may be conscious and explicit, as it is in the case of traditional organizations that expect adherents to prioritize communication from fellow members [71]. However, subconscious biases also impact how individuals prioritize their attention to information sources [72]. Our simulation does not distinguish between conscious and unconscious biasing of communication, which have the same observable outcome. Consequently, the simulation results suggest that it may be difficult to discern from network data whether a deliberate act of organizing or mere social affiliation has caused the traits of organization to emerge. This ambiguity may be viewed as a shortcoming of network data, or as further evidence that organization may exist without conscious organizing. As discussed previously, prior CCO research already has found that organizations may emerge before their own members are fully aware of their role [9].

### Applicability beyond simulated scenario

The simulation is predicated on the existence of cognitive biases that are nearly universally experienced by humans, and its implications therefore seem equally broadly applicable. The simulation is further validated by outcomes consistent with observations of group dynamics beyond CCO theory. For example, at steady state the experimental scenario of emerging organization has developed stable heterogeneity among group alliances, with strong links within groups and weak links between them. This mirrors the enduring divisions of human social structure [73, 74]. Our proposed mechanisms thus replicate real-world results that common diffusion models do not, without the unnecessary complexity of adding negative edges to achieve such structuring [75].

An important aspect of our experiment is that it only includes individual goal-driven behavior. Although the agents declare an affiliation, their nascent sense of group identity does not drive any attempt to deliberately build the organization nor disrupt their individual pursuit of maximum personal utility. The simulation therefore is limited to how cognitive biases may spark the initial emergence of organization from a prior state of total isolation. However, once individuals start to become conscious of a sense of group identity, a phase transition may occur and their motives and behavior may change.

Nonetheless, our simulation also hints at how homophily and cascades of reputation may cause leaders to emerge alongside an organization. The emergence of leaders in networks is a field of robust study, and typically incorporates some factor of personality or network structure [76]. We contribute to this discussion by demonstrating how individuals may become the nominal head of a group of co-affiliates, who then support the emergent group identity to avoid appearing in the local minority, without the original figurehead actually exhibiting any traits of leadership nor necessarily even seeking such a position. History, albeit with much more complexity and nuance, bears evidence of real-world analogs to the “passive” leaders of the simulation who become the focus of group identity merely by associating their identity with random messages. For example, modern monarchs often are described as symbolic providers of national identity rather than leaders [77]. We do not mean to suggest that all leadership is an illusion, but rather that the simulation may illustrate the process by which some leaders first gain the prominence to more actively influence group affairs.

The premise of a spontaneous organization, lacking shared objectives, may challenge the appropriateness of some legal constructs. For example, the Racketeer Influenced and Corrupt Organizations (RICO) title of the Organized Crime Control Act of 1970, used in the United States to prosecute organized crime, depends on the determination that an “enterprise” is present [78]. Similarly, many nations make use of the notion of a “terorist organization” in law [79]. The appropriateness of these labels is often debated in academia and court, and it seems that the empirical demonstration of undirected organization may contribute to these discussions, particularly at the edge cases as organizations begin to emerge or are on the verge of disappearance from social significance.

## Conclusion and future research

The most significant contribution of this project is the validation of metrics to measure the emergence of organization from data of communication on a social network. By demonstrating that the four flows of CCO may be observed in a simulated environment that presumes only basic cognitive mechanisms and the ability to affiliate with a social cohort, we have refined the four flows framing of CCO without refuting it. We have reaffirmed the centrality of communication to organization, showing that local variations in communication alone can produce all of the aggregate behaviors associated with organizing. McPhee and Zaug argued that organization would emerge at the intersection of the four flows, and additionally required some element of group identity before true organization could occur [2]. The interrelationships among our proposed metrics and their joint emergence from common mechanisms supports the first of these claims, and that the ability of an agent to co-affiliate is the sole mechanism differentiating the partially-organized from non-organized scenarios supports the second claim. However, by lacking central coordination, cooperation, or any narrative of what group affiliation entails, our simulation pushes the boundaries of the scenarios that McPhee and Zaug portrayed and suggests common mechanisms for the later notion of partial organization [7].

We hope that future research may further validate our metrics using real-world data. CCO theorists have posited that agency and structure mutually constitute one another; individuals and their collective organizations gain social power through relationships and norms that are built through patterns of action [4]. The total lack of apparent organizational agency in our simulation may infer that its collection of cognitive processes provides only the seed of initial organization, a spark that lights this set of paired processes and eventually leads to more sophisticated organizational behavior. We defer theorizing and testing mechanisms for this trajectory to future research. We also leave to future experimentation testing what would be the impact of limiting the overall number of relationships, e.g. the size of an agent’s neighborhood, rather than the number of communication events per turn. This modification seems more aligned with the work of Dunbar [56], and therefore potentially more fundamental to human experience than the modern notion of limited bandwidth to process a seemingly-endless stream of messages. Finally, we note that our simulation reaches a steady-state distribution of agents into stable communities. We hope further exploration may yield what mechanisms cause organizations to become more fluid, causing not just the emergence but also the decline of partial organizations.

## Supporting information

S1 Appendix Simulation code. (PDF)

## References

[1] Schoeneborn D, Kuhn TR, Kärreman D. The communicative constitution of organization, organizing, and organizationality. Organization Studies. 2019;40(4):475–496. doi: 10.1177/0170840618782284

[2] McPhee RD, Zaug P. The communicative constitution of organizations: A framework for explanation. Electronic Journal of Communication. 2000;10(1):1–16.

[3] Schwing KM, Spitaletta J, Pitt J. A mathematical interpretation of the communicative constitution of organizations. Dynamics of Asymmetric Conflict. 2022;15(2):165–188. doi: 10.1080/17467586.2021.2008462

[4] Bisel R. A Communicative Ontology of Organization? A Description, History, and Critique of CCO Theories for Organization Science. Management Communication Quarterly. 2010;24(1):124–131. doi: 10.1177/0893318909351582

[5] Sillince JA. Can CCO theory tell us how organizing is distinct from markets, networking, belonging to a community, or supporting a social movement? Management Communication Quarterly. 2010;24(1):132–138. doi: 10.1177/0893318909352022

[6] McPhee RD, Poole MS, Iverson J. Structuration Theory. In: Putnam LL, Mumby DK, editors. The SAGE Handbook of Organizational Communication: Advances in Theory, Research, and Methods. SAGE Publications; 2014. p. 75–99.

[7] Ahrne G, Brunsson N. Organization outside organizations: The significance of partial organization. Organization. 2011;18(1):83–104. doi: 10.1177/1350508410376256

[8] Bisel R. Organizational Moral Learning: A Communication Approach. Routledge; 2017.

[9] Wilhoit ED, Kisselburgh LG. Collective action without organization: The material constitution of bike commuters as collective. Organization Studies. 2015;36(5):573–592. doi: 10.1177/0170840614556916

[10] Cooren F, Kuhn T, Cornelissen JP, Clark T. Communication, organizing and organization: An overview and introduction to the special issue. Organization Studies. 2011;32(9):1149–1170. doi: 10.1177/0170840611410836

[11] Grothe-Hammer M. Organization without actorhood: Exploring a neglected phenomenon. European Management Journal. 2019;37(3):325–338. doi: 10.1016/j.emj.2018.07.009

[12] King BG, Felin T, Whetten DA. Perspective—Finding the organization in organizational theory: A meta-theory of the organization as a social actor. Organization Science. 2010;21(1):290–305. doi: 10.1287/orsc.1090.0443

[13] Blaschke S, Schoeneborn D, Seidl D. Organizations as networks of communication episodes: Turning the network perspective inside out. Organization Studies. 2012;33(7):879–906. doi: 10.1177/0170840612443459

[14] Mika P. Ontologies are us: A unified model of social networks and semantics. Journal of Web Semantics. 2007;5(1):5–15. doi: 10.1016/j.websem.2006.11.002

[15] Fortunato S. Community detection in graphs. Physics Reports. 2010;486(3):75–174. doi: 10.1016/j.physrep.2009.11.002

[16] Luo J, Magee CL. Detecting evolving patterns of self-organizing networks by flow hierarchy measurement. Complexity. 2011;16(6):53–61. doi: 10.1002/cplx.20368

[17] El-Moussaoui M, Agouti T, Tikniouine A, El Adnani M. A comprehensive literature review on community detection: Approaches and applications. Procedia Computer Science. 2019;151:295–302. doi: 10.1016/j.procs.2019.04.042

[18] Sangkaran T, Abdullah A, JhanJhi N. Criminal network community detection using graphical analytic methods: A survey. EAI Endorsed Transactions on Energy Web. 2020;7(26):e5.

[19] Rostami A, Mondani H, Liljeros F, Edling C. Criminal organizing applying the theory of partial organization to four cases of organized crime. Trends in Organized Crime. 2018;21(4):315–342. doi: 10.1007/s12117-017-9315-6

[20] Kim J, Hastak M. Social network analysis: Characteristics of online social networks after a disaster. International Journal of Information Management. 2018;38(1):86–96. doi: 10.1016/j.ijinfomgt.2017.08.003

[21] Ford KC, Veletsianos G, Resta P. The Structure and Characteristics of #PhDChat, an Emergent Online Social Network. Journal of Interactive Media in Education. 2014;. doi: 10.5334/2014-08

[22] Lusseau D. The emergent properties of a dolphin social network. Proceedings of the Royal Society of London Series B: Biological Sciences. 2003;270:S186–S188. doi: 10.1098/rsbl.2003.0057 14667378 PMC1809954

[23] Hazy JK. Leading large: Emergent learning and adaptation in complex social networks. International Journal of Complexity in Leadership and Management. 2012;2(1):52–73. doi: 10.1504/IJCLM.2012.050395

[24] Nepusz T, Vicsek T. Hierarchical self-organization of non-cooperating individuals. PloS One. 2013;8(12). doi: 10.1371/journal.pone.0081449 24349070 PMC3859486

[25] Tokita CK, Tarnita CE. Social influence and interaction bias can drive emergent behavioural specialization and modular social networks across systems. Journal of the Royal Society Interface. 2020;17(162). doi: 10.1098/rsif.2019.0564 31910771 PMC7014790

[26] Tasselli S, Kilduff M, Menges JI. The microfoundations of organizational social networks: A review and an agenda for future research. Journal of Management. 2015;41(5):1361–1387. doi: 10.1177/0149206315573996

[27] Marois R, Ivanoff J. Capacity limits of information processing in the brain. Trends in Cognitive Sciences. 2005;9(6):296–305. doi: 10.1016/j.tics.2005.04.010 15925809

[28] Lerman K. Information is not a virus, and other consequences of human cognitive limits. Future Internet. 2016;8(2):21. doi: 10.3390/fi8020021

[29] Miritello G, Lara R, Cebrian M, Moro E. Limited communication capacity unveils strategies for human interaction. Scientific Reports. 2013;3(1):1–7. doi: 10.1038/srep01950 23739519 PMC3674429

[30] Ricci A, Omicini A, Viroli M, Gardelli L, Oliva E. Cognitive stigmergy: Towards a framework based on agents and artifacts. In: Third International Workshop on Environments for Multi-Agent Systems. Springer; 2007. p. 124–140.

[31] Raeder T, Lizardo O, Hachen D, Chawla NV. Predictors of short-term decay of cell phone contacts in a large scale communication network. Social Networks. 2011;33(4):245–257. doi: 10.1016/j.socnet.2011.07.002

[32] Candia C, Jara-Figueroa C, Rodriguez-Sickert C, Barabási AL, Hidalgo CA. The universal decay of collective memory and attention. Nature Human Behaviour. 2019;3(1):82–91. doi: 10.1038/s41562-018-0474-5 30932052

[33] Arena G, Mulder J, Leenders RTA. A Bayesian semi-parametric approach for modeling memory decay in dynamic social networks. Sociological Methods & Research. 2022;. doi: 10.1177/00491241221113875

[34] Burt RS. Attachment, decay, and social network. Journal of Organizational Behavior: The International Journal of Industrial, Occupational and Organizational Psychology and Behavior. 2001;22(6):619–643. doi: 10.1002/job.106

[35] Roberts SB, Dunbar RI. Managing relationship decay. Human Nature. 2015;26(4):426–450. 26489745 10.1007/s12110-015-9242-7 PMC4626528

[36] Butts CT. A relational event framework for social action. Sociological Methodology. 2008;38(1):155–200. doi: 10.1111/j.1467-9531.2008.00203.x

[37] Marsh L, Onof C. Stigmergic epistemology, stigmergic cognition. Cognitive Systems Research. 2008;9(1):136–149. doi: 10.1016/j.cogsys.2007.06.009

[38] Heylighen F. Stigmergy as a universal coordination mechanism I: Definition and components. Cognitive Systems Research. 2016;38:4–13. doi: 10.1016/j.cogsys.2015.12.007

[39] Bindra S, Sharma D, Parameswar N, Dhir S, Paul J. Bandwagon effect revisited: A systematic review to develop future research agenda. Journal of Business Research. 2022;143:305–317. doi: 10.1016/j.jbusres.2022.01.085

[40] Barnfield M. Think twice before jumping on the bandwagon: Clarifying concepts in research on the bandwagon effect. Political Studies Review. 2020;18(4):553–574. doi: 10.1177/1478929919870691

[41] Nadeau R, Cloutier E, Guay JH. New evidence about the existence of a bandwagon effect in the opinion formation process. International Political Science Review. 1993;14(2):203–213. doi: 10.1177/019251219301400204

[42] Chao WM, Li TY. Simulating riot for virtual crowds with a social communication model. In: International Conference on Computational Collective Intelligence. Springer; 2011. p. 419–427.

[43] Gavious A, Mizrahi S. A continuous time model of the bandwagon effect in collective action. Social Choice and Welfare. 2001;18(1):91–105. doi: 10.1007/s003550000061

[44] Guilbeault D, Becker J, Centola D. Complex Contagions: A Decade in Review. In: Lehmann S, Ahn YY, editors. Complex Spreading Phenomena in Social Systems: Influence and Contagion in Real-World Social Networks. Springer; 2018. p. 3–25.

[45] Centola D. How Behavior Spreads: The Science of Complex Contagions. Princeton University Press; 2018.

[46] McPherson M, Smith-Lovin L, Cook JM. Birds of a feather: Homophily in social networks. Annual Review of Sociology. 2001;27(1):415–444. doi: 10.1146/annurev.soc.27.1.415

[47] Fu F, Nowak MA, Christakis NA, Fowler JH. The evolution of homophily. Scientific Reports. 2012;2(1):1–6. doi: 10.1038/srep00845 23150792 PMC3496167

[48] Kossinets G, Watts DJ. Origins of homophily in an evolving social network. American Journal of Sociology. 2009;115(2):405–450. doi: 10.1086/599247

[49] Jackson MO, López-Pintado D. Diffusion and contagion in networks with heterogeneous agents and homophily. Network Science. 2013;1(1):49–67. doi: 10.1017/nws.2012.7

[50] Aral S, Muchnik L, Sundararajan A. Engineering social contagions: Optimal network seeding in the presence of homophily. Network Science. 2013;1(2):125–153. doi: 10.1017/nws.2013.6

[51] Shalizi CR, Thomas AC. Homophily and contagion are generically confounded in observational social network studies. Sociological Methods & Research. 2011;40(2):211–239. doi: 10.1177/0049124111404820 22523436 PMC3328971

[52] Aral S, Muchnik L, Sundararajan A. Distinguishing influence-based contagion from homophily-driven diffusion in dynamic networks. Proceedings of the National Academy of Sciences. 2009;106(51):21544–21549. doi: 10.1073/pnas.0908800106 20007780 PMC2799846

[53] Boomgaard G, Lavitt F, Treur J. Computational analysis of social contagion and homophily based on an adaptive social network model. In: International Conference on Social Informatics. Springer; 2018. p. 86–101.

[54] Treur J. Mathematical analysis of the emergence of communities based on coevolution of social contagion and bonding by homophily. Applied Network Science. 2019;4(1):1–30. doi: 10.1007/s41109-019-0130-7

[55] Michener JA. The Bridge at Andau. Fawcett Publications; 1957.

[56] Dunbar RI. The social brain hypothesis. Evolutionary Anthropology: Issues, News, and Reviews. 1998;6(5):178–190. doi: 10.1002/(SICI)1520-6505(1998)6:5<178::AID-EVAN5>3.0.CO;2-8

[57] Dunbar R. Culture, Honesty, and the Freerider Problem. In: Dunbar R, Knight C, Power C, editors. The Evolution of Culture. Rutgers University Press; 1999. p. 194–213.

[58] El Karhili N, Hendry J, Kackowski W, El Damanhoury K, Dicker A, Winkler C. Islamic/State: Daesh’s Visual Negotiation of Institutional Positioning. Journal of Media and Religion. 2021;20(2):1–26. doi: 10.1080/15348423.2021.1930813

[59] Schwing KM, Spitaletta J, Pitt J. Measuring the competitive communicative constitution of insurgencies and their opponents. Dynamics of Asymmetric Conflict. 2023;16(2):71–96. doi: 10.1080/17467586.2023.2218888

[60] Javed MA, Younis MS, Latif S, Qadir J, Baig A. Community detection in networks: A multidisciplinary review. Journal of Network and Computer Applications. 2018;108:87–111. doi: 10.1016/j.jnca.2018.02.011

[61] Ogburn EL. Challenges to estimating contagion effects from observational data. In: Lehmann S, Ahn YY, editors. Complex Spreading Phenomena in Social Systems: Influence and Contagion in Real-World Social Networks. Springer; 2018. p. 47–64.

[62] Simon CP, Blume L, et al. Mathematics for economists. vol. 7. Norton New York; 1994.

[63] Bianconi G, Darst RK, Iacovacci J, Fortunato S. Triadic closure as a basic generating mechanism of communities in complex networks. Physical Review E. 2014;90(4):042806. doi: 10.1103/PhysRevE.90.042806 25375548

[64] Opsahl T, Panzarasa P. Clustering in weighted networks. Social Networks. 2009;31(2):155–163. doi: 10.1016/j.socnet.2009.02.002

[65] Grothe-Hammer M. Membership and contributorship in organizations: An update of modern systems theory. Systems Research and Behavioral Science. 2020;37(3):482–495. doi: 10.1002/sres.2683

[66] Banks J, Brooks J, Cairns G, Davis G, Stacey P. On Devaney’s definition of chaos. The American Mathematical Monthly. 1992;99(4):332–334. doi: 10.1080/00029890.1992.11995856

[67] Taylor JR, Van Every EJ. The emergent organization: Communication as its site and surface. Routledge; 1999.

[68] Helbing D. Social Self-Organization: Agent-Based Simulations and Experiments to Study Emergent Social Behavior. Springer; 2012.

[69] Dietz H. Ethnicity, Integration and the Military. Routledge; 2021.

[70] Putnam RD. Bowling alone: The Collapse and Revival of American Community. Simon and Schuster; 2000.

[71] Pluut H, Flestea AM, Curşeu PL. Multiple team membership: A demand or resource for employees? Group Dynamics: Theory, Research, and Practice. 2014;18(4):333. doi: 10.1037/gdn0000016

[72] Appiah O, Knobloch-Westerwick S, Alter S. Ingroup favoritism and outgroup derogation: Effects of news valence, character race, and recipient race on selective news reading. Journal of Communication. 2013;63(3):517–534. doi: 10.1111/jcom.12032

[73] Dakin R, Ryder TB. Reciprocity and behavioral heterogeneity govern the stability of social networks. Proceedings of the National Academy of Sciences. 2020;117(6):2993–2999. doi: 10.1073/pnas.1913284117 31980520 PMC7022202

[74] Karsai M, Perra N, Vespignani A. Time varying networks and the weakness of strong ties. Scientific Reports. 2014;4(1):1–7. doi: 10.1038/srep04001 24510159 PMC3918922

[75] Stadtfeld C, Takács K, Vörös A. The emergence and stability of groups in social networks. Social Networks. 2020;60:129–145. doi: 10.1016/j.socnet.2019.10.008

[76] Carter DR, DeChurch LA, Braun MT, Contractor NS. Social network approaches to leadership: An integrative conceptual review. Journal of Applied Psychology. 2015;100(3):597. doi: 10.1037/a0038922 25798551

[77] Bix HP. Inventing the “Symbol Monarchy” in Japan, 1945-52. Journal of Japanese Studies. 1995;21(2):319–363. doi: 10.2307/133011

[78] Blakey GR, Gettings B. Racketeer Influenced and Corrupt Organizations (RICO): Basic Concepts-Criminal and Civil Remedies. Temple Law Quarterly. 1980;53:1009–1048.

[79] Golder B, George W. What is ‘terrorism’? Problems of legal definition. University of New South Wales Law Journal. 2004;27:270.

