# Supplementary material for: Measuring the communicative constitution of organization as network formation
Source: PLoS One. 2024 Apr 9;19(4):e0300399. doi: 10.1371/journal.pone.0300399 (PMC11003641; doi:10.1371/journal.pone.0300399)
Supplement: S1 Appendix — (PDF) [file pone.0300399.s001.pdf]

Appendix: Simulation code

```
import random
import statistics


class Agent:
    def __init__(self,num):
        #The initial location of the agent is random
        self.x=random.randint(0,mapLength-1)
        self.y=random.randint(0,mapLength-1)
        #Agent initially is affiliated with itself
        self.affiliation=0
        #Channel capacity initially is set to maximum
        self.capacity=bandwidth
        #Traits (V) are set randomly
        self.traits=[random.randint(1,maxTrait) for x in range(
            numTrait)]
        self.neighbors=[0 for x in range(popSize)]
        self.oldNeighborhood=[num]
        self.bids=[0 for x in range(popSize)]
        self.currentRole=num
        self.favTrait=random.randint(0,(numTrait-1))

    #Wander is the random walk of the agents
    def Wander(self):
        self.direction=random.randint(0,3)
        if(self.direction==0):
            self.x=self.x+1
        if(self.direction==1):
            self.y=self.y+1
        if(self.direction==2):
            self.x=self.x-1
```

```
        if(self.direction==3):
            self.y=self.y-1
        #World is a torus
        if(self.x==mapLength):
            self.x=0
        if(self.y==mapLength):
            self.y=0
        if(self.x==-1):
            self.x=(mapLength-1)
        if(self.y==-1):
            self.y=(mapLength-1)

#Global variables
#Number of turns
totalT=50
#Length of one side of the world
mapLength=10
#Maximum number of communication events that an agent can
    participate in per turn
bandwidth=6
maxTrait=5
numTrait=5
popSize=150
#Rate of communication edge decay per turn
d=0.8
#Percent of gap between ideal and actual neighbor edge strength
     that is filled for co-affiliates
b=1
#Threshold for inclusion in neighborhood
nT=0.5
#Toggle whether experimental or control scenario
```

```
experimental=False

#Number of random trials per experiment
trials=50

#Average number of times an agent switched roles across the
    experiment
BigRoleChanges=[0 for x in range(totalT)]
#Average number of times an agent switched affiliation across
    the experiment
BigAffilChanges=[0 for x in range(totalT)]
#Average number of changes of neighborhood membership between
    turns across the experiment
BigNeighChanges=[0 for x in range(totalT)]
#Average conditional probability of a neighborhood triangle,
    given two legs of the triangle across the experiment
BigProbTriangle=[0 for x in range(totalT)]
#Average variance of all edges across the experiment
BigAvgVarAll=[0 for x in range(totalT)]
#Average total utility of population across the experiment
BigTotalUtil=[0 for x in range(totalT)]

for trial in range(trials):

    #Dependent variables
    #Number of times an agent switched roles
    roleChanges=[0 for x in range(totalT)]
    #Number of times an agent switched affiliation
    affilChanges=[0 for x in range(totalT)]
    #Number of changes of neighborhood membership between turns
    neighChanges=[0 for x in range(totalT)]
```

```
#Conditional probability of a neighborhood triangle, given
    two legs of the triangle
probTriangle=[0 for x in range(totalT)]
#Average variance of all edges
avgVarAll=[0 for x in range(totalT)]
#Total utility score of all agents
totalUtil=[0 for x in range(totalT)]

#Initialize population
population=[Agent(i) for i in range(popSize)]
roles=[[random.randint(1,maxTrait) for j in range(numTrait)
    ] for i in range(popSize)]

#Turn loop
for turn in range(totalT):

    #Variables that reset each turn
    yesTri=0
    noTri=0
    neighVar=0
    allVar=0
    utils=[]
    for i in range(popSize):
        population[i].capacity=bandwidth

    #Agents take random walk
    for i in range(popSize):
        population[i].Wander()

    #Agents meet new neighbors
    #Note that an agent will always be a neighbor with
```

```
    itself with a strength of 1
for i in range(popSize):
    myX=population[i].x
    myY=population[i].y
    for j in range(popSize):
        if((myX==population[j].x)and(myY==population[j
            ].y)):
            population[i].neighbors[j]=1

#Agents chatter while capacity exists anywhere in the
    network
totalCapacity=0
for i in range(popSize):
    totalCapacity=totalCapacity+population[i].capacity
while(totalCapacity>0):
    #Message begins at random agent
    i=random.randint(0,popSize-1)
    comList=[i]
    #Message adopts affiliation of its origin
    comAffil=population[i].affiliation
    #A list records what agents received (or self-
        generated) the message
    comRec=[]
    while(len(comList)>0):
        #Message has a bias toward first nodes to have
            received it
        x=comList.pop(0)
        if(experimental==True):
            #If a receiving agent is of a different
                affiliation, the message may prompt it
                to convert
```

```
if(population[x].affiliation!=comAffil):
    oldScore=0
    newScore=0
    #Search across population
    for j in range(popSize):
        #Filter against self-conversion
        if(x!=j):
            #The agent only considers the
                affiliation of its
                neighbors.
            if(population[x].neighbors[j]>
                nT):
                fT=population[x].favTrait
                #The agent evaluates
                    keeping its current
                    affiliation
                if(population[x].
                    affiliation==population
                    [j].affiliation):
                    oldScore=oldScore+4-abs
                        (population[x].
                        traits[fT]-
                        population[j].
                        traits[fT])
                #The agent evaluates
                    switching to the
                    affiliation of the
                    received message
                if(comAffil==population[j].
                    affiliation):
                    newScore=newScore+4-abs
```

```
                                    (population[x].
                                    traits[fT]-
                                    population[j].
                                    traits[fT])
                    #The agent potentially changes
                        affiliation
                    if(newScore>oldScore):
                        population[x].affiliation=comAffil
                        affilChanges[turn]=affilChanges[
                            turn]+1

        #Message will only propagate from an agent that
            has capacity
        if(population[x].capacity>0):
            #Decrement capacity of sending agent. Note
                that multiple agents may receive a
                message sent once.
            population[x].capacity=population[x].
                capacity-1
            totalCapacity=totalCapacity-1
            if(not(x in comRec)):
                comRec.append(x)
            #Attempt to pass message to all other
                agents, in random order
            for j in random.sample(range(popSize),
                popSize):
                #Filter against echoes
                if(not(j in comRec)):
                    m=random.random()
                    bond=population[x].neighbors[j]
                    if(population[x].affiliation==
```

```
                    population[j].affiliation):
                     bond=bond+(1-bond)*b
                 if((bond>m)and(population[j].
                    capacity>0)):
                     #Add receiving agent to list so
                         that message can continue
                        to propagate
                     comList.append(j)
                     #Decrement capacity of
                        receiving agent
                     population[j].capacity=
                        population[j].capacity-1
                     totalCapacity=totalCapacity-1
                     #Record that agent received
                        message
                     comRec.append(j)
                     #Strength of network edge
                        reverts to 1 with
                        successful communication
                     population[x].neighbors[j]=1
                     population[j].neighbors[x]=1
#Agents place bids for roles of their neighbors
for i in random.sample(range(popSize),popSize):
    #Reset variables for each agent
    neighborhood=[]
    allEdges=[]
    scores=[]
    #Build list of indices of neighbors and list of
        their link strengths
    for j in range(popSize):
        if(population[i].neighbors[j]>nT):
```

```
            neighborhood.append(j)
    #Take advantage of this list to track how
        neighborhoods change
    neighChanges[turn]=neighChanges[turn]+len(set(
        neighborhood).symmetric_difference(set(
        population[i].oldNeighborhood)))
    population[i].oldNeighborhood=neighborhood
    #Count number of triangles among neighbors
    for j in range(len(neighborhood)):
        for k in range(len(neighborhood)):
            if((i!=j)and(i!=k)and(j!=k)):
                if(population[j].neighbors[k]>nT):
                    yesTri=yesTri+1
                if(population[j].neighbors[k]<=nT):
                    noTri=noTri+1

    #Measure variance of all links
    allEdges=population[i].neighbors.copy()
    #Remove the link to self
    allEdges.remove(1)
    allVar=allVar+statistics.pvariance(allEdges)

    #Build list of current roles of neighbors
    for j in range(len(neighborhood)):
        t=population[neighborhood[j]].currentRole
        score=0
        for k in range(numTrait):
            score=score+roles[t][k]*population[i].
                traits[k]
        z=[score,t]
        scores.append(z)
```

```
        #Bids for tied scores will be in random order
        random.shuffle(scores)
        scores.sort(reverse=True, key=lambda x: x[0])
        #Place bids
        population[i].bids=[0 for x in range(popSize)]
        w=popSize
        for j in range(len(scores)):
            population[i].bids[scores[j][1]]=w
            w=w-1

    #Resolving bids to assign roles, in random order
    for t in random.sample(range(popSize),popSize):
        highBidder=[0]
        highBid=population[0].bids[t]
        for i in range(popSize-1):
            if(population[i+1].bids[t]>highBid):
                highBidder=[i+1]
                highBid=population[i+1].bids[t]
            if(population[i+1].bids[t]==highBid):
                highBidder.append((i+1))
        highBidder=random.choice(highBidder)
        #Count if a role change occurs
        if(population[highBidder].currentRole!=t):
            roleChanges[turn]=roleChanges[turn]+1
        population[highBidder].currentRole=t
        population[highBidder].bids=[-1 for i in range(
            popSize)]

    #Network links decay
    for i in range(popSize):
        for j in range(popSize):
```

```
                population[i].neighbors[j]=d*population[i].
                    neighbors[j]

            #Record network data
            #Compute triangle probability
            probTriangle[turn]=yesTri/(yesTri+noTri)
            #Compute average variance of all edges
            avgVarAll[turn]=allVar/popSize
            #Compute utiliy of all agents
            for i in range(popSize):
                t=population[i].currentRole
                score=0
                for k in range(numTrait):
                    score=score+roles[t][k]*population[i].traits[k]
                utils.append(score)
                totalUtil[turn]=totalUtil[turn]+score

        for i in range(totalT):
            BigAffilChanges[i]=BigAffilChanges[i]+affilChanges[i]
            BigRoleChanges[i]=BigRoleChanges[i]+roleChanges[i]
            BigNeighChanges[i]=BigNeighChanges[i]+neighChanges[i]
            BigProbTriangle[i]=BigProbTriangle[i]+probTriangle[i]
            BigAvgVarAll[i]=BigAvgVarAll[i]+avgVarAll[i]
            BigTotalUtil[i]=BigTotalUtil[i]+totalUtil[i]

    #Average each output variable over total number of trials
    for i in range(totalT):
        BigAffilChanges[i]=BigAffilChanges[i]/trials
        BigRoleChanges[i]=BigRoleChanges[i]/trials
        BigNeighChanges[i]=BigNeighChanges[i]/trials
        BigProbTriangle[i]=BigProbTriangle[i]/trials
```

```
        BigAvgVarAll[i]=BigAvgVarAll[i]/trials
        BigTotalUtil[i]=BigTotalUtil[i]/trials

print("Affiliation Changes: ",BigAffilChanges)
print("Role Changes: ",BigRoleChanges)
print("Neighborhood Changes: ",BigNeighChanges)
print("Probability Triangle: ",BigProbTriangle)
print("All Variance: ",BigAvgVarAll)
print("Total Utility: ",BigTotalUtil)
```
